# Elimination of SHIV Infected Cells by Combinations of Bispecific HIVxCD3 DART^®^ Molecules

**DOI:** 10.3389/fimmu.2021.710273

**Published:** 2021-08-13

**Authors:** Marina Tuyishime, Amir Dashti, Katelyn Faircloth, Shalini Jha, Jeffrey L. Nordstrom, Barton F. Haynes, Guido Silvestri, Ann Chahroudi, David M. Margolis, Guido Ferrari

**Affiliations:** ^1^ Department of Surgery, Duke University Medical Center, Durham, NC, United States; ^2^ Department of Pediatrics, Emory University, Atlanta, GA, United States; ^3^ Research Department, MacroGenics, Rockville, MD, United States; ^4^ Duke Human Vaccine Institute, Durham, NC, United States; ^5^ Department of Medicine, Duke University Medical Center, Durham, NC, United States; ^6^ Department of Immunology, Duke University Medical Center, Durham, NC, United States; ^7^ Yerkes National Primate Research Center, Emory University, Atlanta, GA, United States; ^8^ Center for Childhood Infections and Vaccines of Children’s Healthcare of Atlanta and Emory University, Atlanta, GA, United States; ^9^ University of North Carolina (UNC) HIV Cure Center, University of North Carolina at Chapel Hill, Chapel Hill, NC, United States; ^10^ Department of Medicine, University of North Carolina at Chapel Hill, Chapel Hill, NC, United States; ^11^ Department of Microbiology and Immunology, University of North Carolina at Chapel Hill, Chapel Hill, NC, United States; ^12^ Department of Epidemiology, University of North Carolina at Chapel Hill, Chapel Hill, NC, United States; ^13^ Department of Molecular Genetics and Microbiology, Duke University Medical Center, Durham, NC, United States

**Keywords:** HIV, bispecific DART molecules, redirected cytotoxicity, cytotoxic T cells, broadly neutralizing antibodies, non-neutralizing antibodies

## Abstract

Bispecific HIVxCD3 DART molecules that co-engage the viral envelope glycoprotein (Env) on HIV-1-infected cells and the CD3 receptor on CD3+ T cells are designed to mediate the cytolysis of HIV-1-infected, Env-expressing cells. Using a novel *ex vivo* system with cells from rhesus macaques (RMs) infected with a chimeric Simian-Human Immunodeficiency Virus (SHIV) CH505 and maintained on ART, we tested the ability of HIVxCD3 DART molecules to mediate elimination of *in vitro*-reactivated CD4+ T cells in the absence or presence of autologous CD8+ T cells. HIVxCD3 DART molecules with the anti-HIV-1 Env specificities of A32 or 7B2 (non-neutralizing antibodies) or PGT145 (broadly neutralizing antibody) were evaluated individually or combined. DART molecule-mediated antiviral activity increased significantly in the presence of autologous CD8+ T cells. In this *ex vivo* system, the PGT145 DART molecule was more active than the 7B2 DART molecule, which was more active than the A32 DART molecule. A triple combination of the DART molecules exceeded the activity of the individual PGT145 DART molecule. Modified quantitative virus outgrowth assays confirmed the ability of the DART molecules to redirect RM CD3+ T cells to eliminate SHIV-infected RM CD4+ T cells as demonstrated by the decreased propagation of *in vitro* infection by the infected cells pre-incubated with DART molecules in presence of effector CD8+ T cells. While mediating cytotoxic activity, DART molecules did not increase proinflammatory cytokine production. In summary, combination of HIVxCD3 DART molecules that have broadly-neutralizing and non-neutralizing anti-HIV-1 Env specificities can leverage the host immune system for treatment of HIV-1 infection but will require appropriate reactivation of the latent reservoir.

## Introduction

Since the first reported cases of Acquired Immunodeficiency Syndrome (AIDS) in 1981, infection with Human Immunodeficiency Virus type 1 (HIV-1) has been deemed a global and persistent epidemic. The treatment of HIV-1 infection with antiretroviral therapy (ART) has been effective in controlling virus replication, delaying disease progression, and reducing HIV-1 transmission (1). The inability of ART to eradicate HIV-1 infection is primarily due to the establishment of a latent reservoir that cannot be successfully targeted by current therapeutic strategies. Due to the presence of integrated but transcriptionally silent proviral HIV DNA, ART has had limited effects in preventing the elimination of the latent reservoir (2, 3) and achieving HIV-1 remission (4, 5). Due to the slow decay rate of the HIV reservoir, computational data suggest that it might take up to 70 years on ART to completely eradicate the infection (5, 6). Latency Reversing Agents (LRAs) have been identified and used to induce proviral transcription in latently infected cells (7–10) with subsequent expression of viral antigens on the cell surface that can be targeted by HIV-1-specific antibodies (Abs).

Broadly neutralizing anti-HIV-1 envelope (Env) antibodies (bNAbs) have been shown to reduce viremia, eliminate sensitive viruses in chronically infected individuals, delay virus rebound (11–17) and provide protection in non-human primate studies (18–25). In addition to virus neutralization, Abs can also eliminate infected cells *via* Fc-mediated functions that include antibody dependent cellular cytotoxicity (ADCC). ADCC activities have been correlated with slow disease progression in HIV-1-infected individuals (26–29). ADCC, driven by bNAbs and non-neutralizing antibodies (non-NAbs), can also mediate killing of cells infected with neutralization resistant viruses (30, 31). Based on these properties of anti-HIV-1 Env Abs, bispecific DART molecules were generated. DART molecules bind to CD3 with one arm and to HIV-1 Env with another, with the ability to engage Env expressed on HIV-1-infected CD4+ T cells, representing the target cells, and CD3 expressed on cytotoxic effector T cells (32). *In vitro* studies show that DART molecules with anti-HIV-1 Env specificities of bNAbs retain the neutralization breadth and potency of the bNAb component (8, 33), and can neutralize newly produced virions post latency reversal. DART and other mAb-based molecules mediated elimination of HIV Env-expressing infected CD4 cell lines and primary human CD4+ T by recruiting *via* anti-CD3 arm cytotoxic CD8+ T from HIV-seronegative and ART-suppressed HIV-seropositive participants (8, 33–36). The original DART molecules had limited *in vivo* pharmacokinetics (bioavailability, solubility, stability, and half-life) compared to traditional Abs (37, 38); therefore, a new molecule was designed to add an Fc region to DART which demonstrated improvement in its half-life (39). One HIVxCD3 DART molecule (MGD014, also known as A32xCD3) is currently in clinical testing in people living with HIV-1 on ART (40).

We previously reported that DART molecules with anti-HIV-1 Env arms based on non-neutralizing A32 (gp120 C1C2, epitope cluster A), non-neutralizing 7B2 (gp41 cluster I) and neutralizing PGT145 (V2 apex) antibodies, redirected human CD8+ T cells to eliminate HIV-1-infected primary autologous CD4+ T cells (41) and latently infected cells from ART-suppressed, HIV-1-seropositive donors that had been reactivated *ex vivo* (33, 34). A triple combination of A32xCD3, 7B2xCD3 and PGT145xCD3 DART molecules, which have anti-CD3 arms that cross-react with rhesus CD3 and were engineered with rhesus Fc domains, was tested *in vivo* in rhesus macaques (RMs) infected with Simian-Human Immunodeficiency Virus expressing the CH505 T/F envelope (SHIV.CH505.375H) (41, 42). However, we reported that the limited sizes of the viral reservoir and the absence of detectable latency reactivation in animals following LRA administration most likely affected our ability to fully assess the anti-viral activity of HIVxCD3 DART molecules (41). In the current study, we further confirm the ability of three DART molecules individually and in combination to eliminate reactivated SHIV.CH505.375H-infected cells using a novel *ex vivo* primary RM system utilizing purified populations of infected target (CD4+) and effector (CD8+) cells. In this autologous system we evaluated whether a combination of DART molecules is more effective than individual DART molecules in mediating clearance of CD4+ T cells isolated from SHIV.CH505.375H-infected RMs by autologous CD8+ T cells. These data support an active role of the DART molecule that will require a more efficient activation of the latent reservoir regardless its size.

## Materials and Methods

### DART Molecules

HIVxCD3 DART molecules are bispecific, Fc-bearing molecules with an anti-HIV-1 Env specificity paired with an anti-human CD3 specificity. The anti-HIV-1 Env specificities were derived from A32 (non-neutralizing antibody (Ab) specific for the cluster A epitope in gp120 C1, C2), 7B2 (non-neutralizing Ab specific for gp41 cluster I) and PGT145 (neutralizing Ab specific for gp120 V2) (33, 34). The anti-human CD3 specificity cross-reacts with rhesus monkey CD3ϵ. The rhesus IgG1 Fc domain utilized for these DART molecules is inactivated for Fc-gamma receptor and complement binding but retains neonatal Fc receptor binding (41). HIVxRSV DART molecules contain anti-respiratory syncytial virus (RSV) specificities instead of anti-CD3 specificities.

### SHIV-Infection of A66 Cells

SHIV.CH505.375H challenge virus stocks, a chimeric simian/human immunodeficiency virus with a SIVmac766 backbone and the clinically relevant HIV-1 transmitted/founder clade C envelope CH505 (41) grown in rhesus PBMCs were titrated to determine the input required for optimal viral gene expression within 72 hours post-infection of A66 cells as measured by intracellular p27 expression, using anti-SIV Gag anti-p27 antibody (WNPRC Immunology Services). A66 cells (provided by James Hoxie, University of Pennsylvania, Philadelphia, PA) are SupT1 cells [non-BC7 variant (43)] that have been stably transfected to express both rhesus CD4 and rhesus CCR5 receptors after knockout of endogenous human CXCR4 and CD4 (44). A66 cells (1 × 10^6^ cells/infection) were incubated with 100 ng/mL p27 of SHIV.CH505.375H for 4 hours at 37°C and 5% CO_2_ in the presence of DEAE-Dextran (10 μg/mL, Sigma Aldrich). The cells were subsequently resuspended at 0.33 × 10^6^/mL and cultured for 3 days in complete medium containing 10 μg/mL DEAE-Dextran. On assay day, infection was monitored by measuring the frequency of cells expressing intracellular p27. The assays performed using the SHIV.CH505.375H-infected target cells were considered reliable if the percentage of viable p27+ target cells on assay day was ≥10%. Assay data generated using infected cells was normalized to the frequency of live target cells positive for intracellular p27.

### Binding of HIVxRSV DART Molecules to SHIV-Infected A66 Cells

SHIV.CH505.375H-infected A66 cells were obtained as described above. Cells incubated in the absence of virus (mock infected) were used as a negative control. Infected and mock-infected cells were washed in PBS, dispensed into 96-well V-bottom plates at 2 x 10^5^ cells/well and incubated with 1 μg/mL of indicated DART molecule for 2 hours at 37 °C. To eliminate CD3 binding, these studies were conducted with HIVxRSV DART molecules in which anti-HIV-1 Env arms were paired with anti-RSV arms instead of anti-CD3 arms. After two washes with 250 μL/well wash buffer (1% FBS in PBS, WB), the cells were stained with vital dye (Live/Dead Fixable Aqua Dead Cell Stain, Invitrogen) to exclude nonviable cells from subsequent analysis. Cells were washed with WB and stained with anti-CD4-PerCP-Cy5.5 (clone Leu-3; BD Biosciences) to a final dilution of 1:20 in the dark for 20 min at room temperature (RT). Cells were then washed again, and permeabilized using Cytofix/Cytoperm (BD Biosciences) for 20 min at 4°C. After wash with 1x Cytoperm wash solution (BD Biosciences), anti-p27 antibody (WNPRC Immunology Services, 1:500 dilution in 1x Cytoperm Solution, BD Biosciences) and a secondary PE-conjugated antibody (goat anti-human Ig Fc-PE, eBioscience, San Diego, CA., final dilution of 1:400) were added to each well and incubated in the dark for 25 min at 4 °C. The secondary anti-human Ig Fc antibody detects the Fc portion of DART molecules bound to the surface of infected cells. Cells were washed 3 times with Cytoperm wash solution and resuspended in PBS-1% paraformaldehyde. The samples were acquired within 24 hours using a BD Fortessa cytometer. A minimum of 50,000 total events was acquired for each analysis. Gates were set to include singlet and live events. The appropriate compensation beads were used to compensate the spill-over signal for the four fluorophores. Data analysis was performed using FlowJo 9.6.6 software (BD Biosciences). Final data represents the PE MFI and frequency of infected cells bound by DART molecules (%p27+/DART+), after normalization by subtracting PE MFI for cells stained with the secondary antibody alone. Assays were repeated twice and the average results are shown.

### *In Vitro* Killing Assays With SHIV-Infected A66 Cells and Human CD8+ T Cells

SHIV.CH505.375H-infected A66 cells, as described above, were used as target cells using a previously described assay (34). Briefly, infected and uninfected target cells were washed in R10 and labelled with a fluorescent target-cell marker (TFL4; OncoImmunin) and a viability marker (NFL1; OncoImmunin) for 15 min at 37°C, as specified by manufacturer. Cells were washed twice in R10 and adjusted to a concentration of 0.2 x 10^6^ cells/mL. On assay day, resting human CD8^+^ effector T cells were isolated by negative selection from PBMCs from healthy donors using a CD8^+^ T cell isolation kit (Miltenyi Biosciences) and were used as effectors with E:T ratio at 33:1. Cells were incubated in the absence or presence of HIVxCD3 DART molecules for 6 hours at the starting concentration of 50 μg/mL with subsequent 6 dilutions at 1:5. Uninfected and infected target cells alone were included as additional controls. Each condition was tested in duplicate. After the incubation period, cells were washed with WB and stained with anti-CD4-PerCP-Cy5.5 (BD Biosciences, clone Leu-3) at a final dilution of 1:20 in the dark for 20 min at RT. After washing with WB, cells were resuspended in 100 μL/well Cytofix/Cytoperm (BD Biosciences), incubated in the dark for 20 min at 4°C, washed in 1x Cytoperm wash solution (BD Biosciences) and co-incubated with anti-SIV Gag p27 antibody (WNPRC Immunology Services) to a final dilution of 1:500 in the dark for 25 min at 4°C. Three washes were performed with Cytoperm wash solution before resuspending the cells in 125 μL PBS-1% paraformaldehyde for acquisition. The samples were acquired within 24 hours using a BD Fortessa cytometer. The appropriate compensation beads were used to compensate the spill-over signal for the four fluorophores. Data analysis was performed using FlowJo 9.6.6 software (BD Biosciences). Mock-infected cells were used to appropriately position live cell p27+/- gates. Redirecting killing activity mediated by the DART molecules was determined by measuring the reduction in the percentage of p27+ cells according to the following formula: % specific killing = [(Frequency of p27 positive cells in wells containing target and effector cells alone − Frequency of p27 positive cells in wells containing target and effector cells plus DART molecules)/Frequency of p27 positive cells in wells containing target and effector cells alone] ×100.

### *Ex Vivo* DART Molecule Treatment of Reactivated, SHIV-Infected RM CD4+ T Cells in the Presence or Absence Of Autologous RM CD8+ T Cells

A novel *ex vivo* assay system was developed to evaluate the ability of HIVxCD3 DART molecules to mediate killing of *in vitro* reactivated CD4+ T cells isolated from SHIV.CH505.375H-infected RMs in the presence or absence of autologous RM CD8+ T cells (**Figure 2A**). PBMCs collected from SHIV-infected RMs at the peak of viremia (week 2 post-infection) were used as the source of SHIV-infected RM CD4+ T cells (targets). PBMCs collected from SHIV-infected RMs on ART (at week 36, when plasma VL levels < 60 copies/mL) were used as the source of CD8+ T cells (effectors). The primary SHIV-infected RM CD4+ T cells were activated for 24 hours with a mixture of antibodies specific for nonhuman primate CD2/CD3/CD28. Reactivated SHIV-infected RM CD4 T cells (1x10^5^ cells/well) were incubated for 48 hours in the absence or presence of autologous CD8 T cells (3x10^3^ cells/well at E:T ratio of 0.03:1) in absence (No DART) or presence of DART molecules individually at 1 µg/mL or combined at 1 µg/mL each. DART molecule-mediated activity was analyzed as % reduction (in absence of autologous CD8+ T cells) and % killing (in presence of autologous CD8+ T cells) by measuring p27 levels in supernatants as following: % p27 reduction/killing = [(p27 ng/mL in wells containing target cells alone − p27 ng/mL in wells containing target and DART molecules (or effector cells, or DART molecules plus effector cells)/p27 ng/mL in wells containing target cells alone] ×100.

### Modified Quantitative Viral Outgrowth Assay

Following the 48-hour incubations of the *ex vivo* killing assays, DART molecules were washed off and A66 feeder cells were added (1x10^6^ cells per well) to allow the propagation of replication competent virus. Cells were passaged with fresh media in the absence of DART molecules every two to three days for a total duration of 9 days. Supernatants from each condition were collected at days 4 and 9 and stored frozen. Supernatants were then thawed and analyzed for SIV Gag p27 levels by ELISA to determine the amounts of SHIV virus that was produced.

### Cytokine and Chemokine Release

Cultures of primary reactivated SHIV-infected CD4+ T cells alone (1x10^5^ cells) or mixed with autologous CD8 T cells (3x10^3^ cells) at E:T ratio of 0.03:1 were incubated without (No DART) or with individual DART molecules at 1 µg/mL or the triple DART molecule combination at 1 µg/mL each for 48 hours. Supernatants were collected and levels of GM-CSF, IFN-γ, IL-1β, IL-6, IL-8, IL-12p40, IL-18, and TNFα determined using a Milliplex^®^ MAP kit (Millipore # HCYTMAG-60K-PX41) on a Luminex MAGPIX™ instrument according to the manufacturer’s protocol. Plates were read on a Sector s600 MSD plate reader and data analyzed using MSD Discovery Workbench analysis software.

## Results

### HIVxCD3 DART Molecule Binding and Specific CD8+ T-Cell Killing of SHIV-Infected A66 Cells

**Figure 1:**
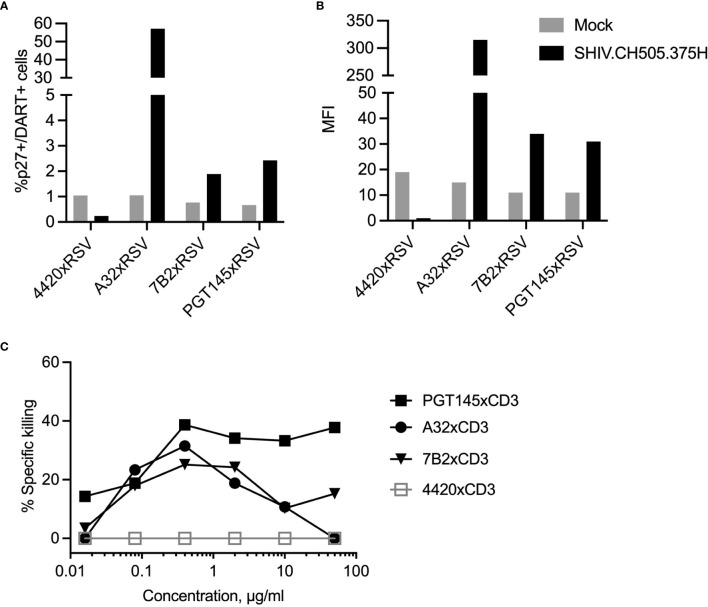
DART molecule binding to SHIV-infected A66 cells and their redirected killing in the presence of human CD8 T cells. The binding of HIVxRSV DART molecules with A32, 7B2 or PGT145 anti-HIV-1 Env specificities to SHIV.CH505.375H-infected A66 cells was evaluated to determine **(A)** frequency of SHIV-infected cells bound by DART molecules (%p27+/DART+) and **(B)** median fluorescent intensity (MFI). The 4420 DART molecule, which contains an anti-fluorescein specificity instead of an anti-HIV-1 Env specificity, was used as the negative control. **(C)** Titration curves represent DART-mediated killing of SHIV.CH505.375H-infected A66 cells as targets (T) by human CD8+ T cells as effectors (E) with E:T ratio of 33:1. The % Specific killing was determined 6 hours post by incubation of T+E+DART molecules measuring the reduction in the percentage of p27+ cells in presence of DART molecules compared to T+E alone.

The ability of the individual DART molecules with A32, 7B2 or PGT145 anti-HIV-1 envelope (Env) specificities to bind to primary human CD4+ T cells activated and infected *in vitro* with HIV-1 transmitted/founder clade C envelope CH505 Infectious Molecular Clone (IMC), and to mediate specific killing by autologous human CD8+ T cells was previously reported (41). Here we first analyzed the ability of the DART molecules to bind to and to redirect human CD8+ T cells to kill SHIV.CH505.375H-infected A66 cells, a human SupT1 cell line modified to stably express RM CD4 and CCR5 receptors. To specifically interrogate binding to CH505 Env on the surface of the SHIV-infected A66 cells, variant DART molecules were generated with anti-HIV-1 Env arms paired with anti-respiratory syncytial virus (anti-RSV) arms instead of anti-CD3 arms. All three HIVxRSV DART molecules demonstrated binding to the SHIV.CH505.375H-infected A66 cells as shown by the frequency (%p27+/DART+) and median fluorescent intensity (MFI) of infected cells bound by the DART molecules (**Figures 1A, B**). The frequency of SHIV.CH505.375H-infected A66 cells bound by A32xRSV, 7B2xRSV or PGT145xRSV was 56%, 2% or 2.5% with MFI of 310, 35 or 30, respectively. Despite the variations in binding, all 3 HIVxCD3 DART molecules mediated specific killing of the SHIV.CH505.375H-infected A66 cells by primary human CD8+ T cells. The killing activity of HIVxCD3 DART molecule, which requires binding to both target and effector cells, occurs at EC_50_ values that range from 1-10 ng/mL (33). Only a small fraction of the binding sites on targets and effectors need to be occupied to enable the formation of immunological synapses that lead to target cell killing. This is consistent with the small number of interactions required for cytotoxic synapses between CTLs and peptide-MHC complexes (45). Thus, dose-dependent effect was observed for specific killing by the individual DART molecules (**Figure 1C**). We chose 1 µg/mL of each DART molecule that shows 20% (A32xCD3), 20% (7B2xCD3) and 40% (PGT145xCD3) specific killing against SHIV.CH505.375H-infected A66 cells and hypothesized that combination of DART molecules each at 1µg/mL will lead to an increase in specific killing compared to individual DART molecules. No binding or specific killing was observed with negative control DART, 4420xRSV, where the anti-HIV arm is substituted for anti-fluorescein.

### *Ex Vivo* Study Design

**Figure 2:**
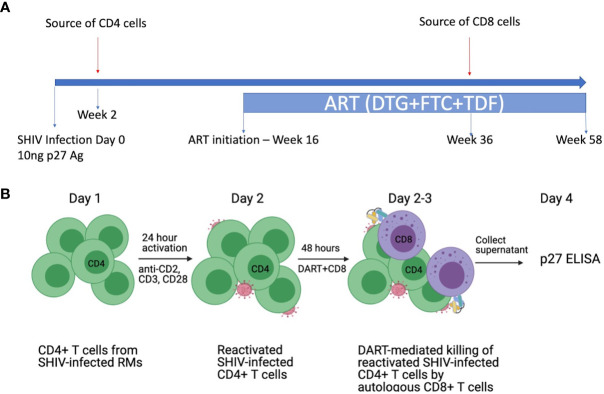
*Ex vivo* killing assays with SHIV-infected RM CD4+ T cells and autologous RM CD8+ T cells. **(A)** Schematic of the *in vivo* study. Rhesus macaques (RMs) were infected intravenously (i.v.) with SHIV.CH505.375H at a dose of 10 ng of SIV Gag p27 antigen (41). SHIV.CH505.375H is a chimeric simian/human immunodeficiency virus with SIVmac backbone and HIV-1 transmitted/founder clade C envelope CH505. The RMs were treated with an ART regimen consisting of tenofovir (TDF: 5.1 mg/kg), emtricitabine (FTC: 40 mg/kg), and dolutegravir (DTG: 2.5 mg/kg) administered subcutaneously daily from week 16 post-infection (p.i.) to the end of the study. PBMCs were collected from the SHIV-infected RMs at week 2 post-infection (prior to ART) to isolate SHIV-infected CD4+ T cells (targets) and at week 36 post-infection (while on ART) to isolate CD8+ T cells (effectors). **(B)** Design of the *ex vivo* killing assays. Primary CD4+ T cells from SHIV.CH505.375H-infected RMs were isolated and activated *in vitro* for 24 hours with antibodies specific for nonhuman primate CD2/CD3/CD28. Activated SHIV-infected RM CD4+ T cells were cultured for 48 hours alone or with autologous RM CD8+ T cells at an E:T ratio of 0.03:1 in the absence or presence of DART molecules. The % killing of the SHIV-infected RM CD4+ T cells was determined by measuring the reduction in supernatant SIV Gag p27 levels compared to CD4+ T cells alone.

To evaluate DART molecule-mediated killing of RM CD4+ T cells collected from SHIV.CH505.375H-infected animals, we developed a novel primary *ex vivo* assay system. PBMCs collected from SHIV.CH505.375H-infected RMs (41) at the peak of viremia (week 2 post-infection) were used as the source of target SHIV-infected RM CD4+ T cells (**Figure 2A**). This time point was chosen because we expected it to provide the highest frequency of circulating infected CD4+ T cells capable of expressing Env on their surface upon reactivation (46) and, thus, represent the most optimal targets for recognition by DART molecules. PBMCs collected from RMs on ART at week 36, when plasma viral load (VL) levels were < 60 copies/mL, were used as the source of effectors CD8+ T cells (41). This time point was chosen to recapitulate pre-clinical studies and clinical trials with administration of DART molecules that rely on post-ART functionality of cytotoxic CD8+ T cells to eliminate reactivated Env-expressing targets (47–49). Primary RM CD4+ T cells were isolated and *in vitro* activated for 24 hours with anti-CD2/CD3/CD28 non-human primate (NHP) Abs and then cultured for 48 hours by themselves or with autologous RM CD8+ T cells at an effector to target (E:T) ratio of 0.03:1 in the absence or presence of DART molecules. The experimental design is shown schematically in **Figure 2B**. This novel system is designed to reflect the diversity of target cells infected by virus isolates circulating *in vivo* and allow evaluation of autologous target and effector cell interactions mediated by DART molecules.

### Virologic Assessment of Primary RM CD4+ T Cells

**Figure 3:**
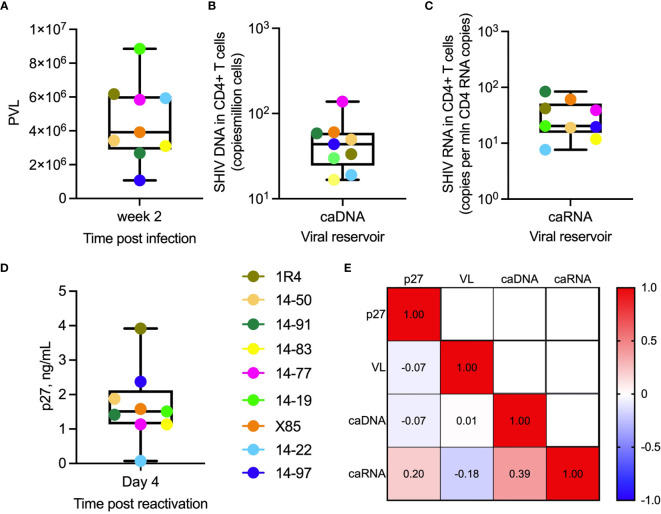
Virologic parameters. **(A)** Plasma viral load (SHIV RNA copies/mL) in individual animals (indicated by their ID designations) following 2 weeks post infection with SHIV.CH505.375H. **(B)** Cell-associated viral DNA (caDNA) and **(C)** cell-associated viral RNA (caRNA) prior to ART initiation at week 16 post-infection. **(D)** SIV Gag p27 levels in supernatants of primary RM CD4+ T cells collected at week 2 post-infection, activated for 24 hours *in vitro* with anti-CD2, CD3, and CD28 antibodies and cultures for 3 days in fresh media. **(A–D)** In the box plots, horizontal lines within boxes denote median values, ends of boxes denote 25^th^ and 75^th^ percentiles, and lines outside of boxes denote minimum and maximum values. **(E)** Spearman correlation analysis of p27 levels in supernatants following *in vitro* activation of RM CD4+ T cells, peak plasma viral load, cell-associated viral RNA, and cell-associated viral DNA. Values are R^2^ values.

The 9 animals in this *ex vivo* study, which were selected based on availability of cryopreserved cellular samples, had peak plasma viral loads (PVL) ranging from 1.5x10^6^ to 9x10^6^ SHIV RNA copies/ml of plasma [**Figure 3A**, **(** 41 **)**]. All animals had established viral reservoirs which were measured by cell-associated SHIV.CH505.375H DNA and RNA (**Figures 3B, C**). The RNA reservoir was smaller than the DNA reservoir, as expected (50). *In vitro* activation of viral gene expression in primary CD4+ T cells from SHIV.CH505.375H-infected RMs with anti-CD2/CD3/CD28 NHP Abs was monitored by measuring SIV Gag p27 levels in supernatants using p27 ELISA; p27 levels ranged from 0.074 to 3.9 ng/mL (**Figure 3D**). We did not observe significant correlations between p27 level in supernatants from cultures of reactivated cells, PVL, level of cell-associated viral RNA, or level of cell-associated viral DNA, as shown by the heat map in **Figure 3E** (R^2^ values ranged from -0.18 to 0.39, with non-significant p values).

### DART Molecule Treatment of Reactivated, SHIV-Infected RM CD4+ T Cells

**Figure 4:**
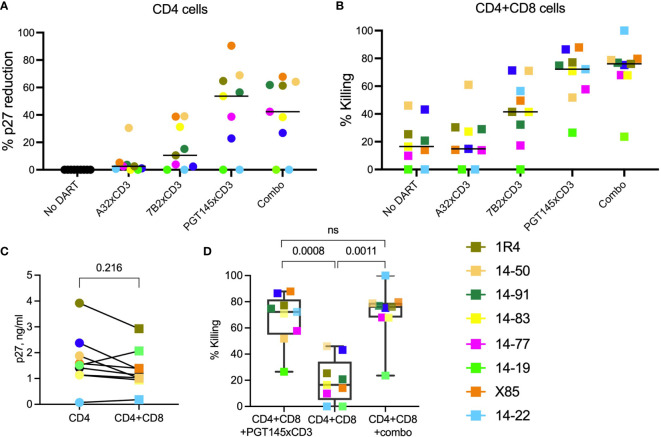
HIVxCD3 DART molecule-mediated killing of SHIV-infected RM CD4+ T cells. *In vitro* activated primary CD4+ T cells from SHIV.CH505.375H-infected RMs were cultured without or with DART molecules in the absence **(A)** or presence **(B)** of autologous CD8+ effector cells at an E:T ratio of 0.03:1. The A32, 7B2 and PGT145 DART molecules at 1 µg/mL were added individually or as a triple combination (Combo) at 1 µg/mL each. The reduction in p27 levels **(A)** and DART-mediated killing **(B)** was analyzed by p27 ELISA in culture supernatants 48 hours post treatment. The circles represent cultures with CD4 cells, squares represent cultures with CD4 and CD8 cells. Each colored symbol represents an individual animal. The horizontal black bars represent median values. Statistical analysis of p27 levels **(C)** or % killing **(D)** between indicated groups was performed using Wilcoxon Test at the significance level of 0.05. NS, not significant.

Supernatant p27 levels in cultures of *in vitro*-reactivated primary CD4+ T cells from SHIV.CH505.375H-infected RMs, incubated without addition of canonical cytotoxic CD8+ T cells, declined noticeably compared to the ‘*No DART*’ control following incubation with A32 DART molecule in 7 of 9 cultures (median reduction 2.5%, range 0.7-30% **Figure 4A**), 7B2 DART molecule in 7 of 9 cultures (median reduction of 10%, range 2.4-39%), PGT145 DART molecule in 7 of 9 cultures (median reduction of 53.6%, range 23-90%), and triple DART combination in 7 of 9 cultures (median reduction of 42%: range 27-68%). Two of the 9 cultures showed no reduction in supernatant p27 level following incubation with the DART molecules. The ranked activities of the DART molecules were PGT145 > 7B2 > A32, and the activity of the triple DART combination was comparable to that of the PGT145 DART molecule alone. Both CD4+ and CD8+ T cell subsets are capable of being redirected to kill HIV-infected Env-expressing target cells, although CD8 cells are generally more potent effectors than CD4 cells (34). Thus, since A32 and 7B2 have non-neutralizing anti-HIV-1 Env specificities, the declines in supernatant p27 observed in presence of A32 and 7B2 DART molecules are interpreted to be due to the killing of Env-expressing SHIV.CH505.375H-infected CD4+ cells by redirected cytotoxic CD4+ T cells. In addition, PGT145 has virus neutralizing activity; therefore, the declines in supernatant p27 mediated by the PGT145 DART molecule may result from both virus neutralization and killing of Env-expressing SHIV-infected CD4+ T cells by redirected cytotoxic CD4+ T cells.

### DART Molecule Treatment of Reactivated, SHIV-Infected RM CD4+ T Cells in the Presence of Autologous RM CD8+ T Cells

We first assessed the elimination of SHIV-infected RM CD4+ T cells by autologous CD8+ T cells at an E:T ratio of 0.03:1 in the absence of DART molecules (**Figure 4B**, *No DART*). We observed a diversity in killing activity in cultures from 7 of 9 animals (median killing 17%, range 10-46%). These results demonstrate that, in this cohort, CD8+ T cells alone are unable to eliminate >50% of the reactivated SHIV-infected RM CD4+ T cells. Next, we analyzed the killing of reactivated SHIV-infected RM CD4+ T cells by autologous CD8 + T cells in the presence of DART molecules. The A32 DART molecule mediated killing in 7 of 9 cultures and increased killing in 6 of 9 cultures compared to CD8+ T cells alone (median killing 15%, range 14-61%). The 7B2 DART molecule mediated killing in 8 of 9 cultures (median killing 41%, range 17-71%), and the PGT145 DART molecule mediated killing in all 9 cultures (median killing 72%, range 27-88%). The ranked activities of the DART molecules were PGT145 > 7B2 > A32. The greater activity of the PGT145 DART molecule observed here is consistent with its greater activity in mediating human CD8+ T-cell killing of HIV-1 CH505 IMC-infected human CD4+ cells *in vitro*, compared to A32 and 7B2 DART molecules, as was previously shown by Dashti et al. (41). It is also consistent with the greater activity of the PGT145 DART molecule in mediating human CD8+ T-cell killing of SHIV.CH505.375H-infected A66 cells (**Figure 1C**). Interestingly, the PGT145 DART molecule was able to mediate low-level killing of reactivated, SHIV-infected RM CD4+ cells from animal 14-19 which exhibited resistance to the activity of the A32 and 7B2 DART molecules. The triple DART molecule combination mediated killing in 9 animals (median killing 76%, range 23.5-99%, **Figure 4B**) and demonstrated an increase in killing activity in 4 of 9 cultures compared to the PGT145 DART molecule alone increasing median killing in those animals from 65% (range 51.8-74.8% by PGT145 DART) to 77.4% (range 68-99% by DART combination). Although DART combination demonstrated an increase in killing activity compared to PGT145 DART alone, the difference did not reach statistical significance (**Figure 4D**).

Despite the limited number of animals, an in-depth analysis of DART molecule-mediated killing activity among the cultures from the 9 animals identifies possible differences in functional profiles. DART combination mediated 99.9% killing of the SHIV-infected RM CD4+ T cells by autologous CD8+ T cells from animal 14-22 however, *in vitro* activation of SHIV-infected RM CD4+ T cells showed the lowest supernatant p27 levels (0.074 ng/mL, **Figure 3C**). These data suggest that low level reactivation of latent infected target cells is sufficient to allow recognition and clearance by DART molecules. However, nearly complete elimination of infected cells could also be due to the lowest numbers of the reactivated SHIV-infected cells present in the *ex vivo* assay. In animal X85, we detected similar levels of % p27 reduction (~90%) when reactivated SHIV-infected CD4+ T cells were cultured with PGT145 DART in the absence (**Figure 4A**) or presence (**Figure 4B**) of CD8 effector cells, suggesting that addition of autologous CD8 cells to these cultures did not increase % killing. These results indicate that CD8 cells from animal X85 could mediate limited cytotoxic activity compared to other animals. In cultures from 5 animals (14-91, 1R4, 14-83, 14-77 and 14-19) the addition of PGT145 DART molecule alone and DART combination drastically increase the % killing of SHIV-infected RM CD4+ T cells by CD8 cells compared to ‘*No DART*’ control, demonstrating the PGT145-driven killing of infected cells in cultures from these animals. The addition of autologous CD8+ T cells did not significantly reduce supernatant p27 levels compared to cultures with CD4 cells alone (**Figure 4C**), while we noticed significantly increased killing of infected cells in presence of PGT145 DART and DART combination compared to CD8 cells alone (**Figure 4D**).

To investigate the differences in cytotoxic activity mediated by CD8+ T cells among animals, we analyzed the CD8+ T cells isolated from PBMCs from each animal using an intracellular cytokine staining (ICS) assay. The cytotoxicity of CD8 cells isolated from each animal did not correlate with production of cytokines (GzB, INFg, TNFa, IL-2) post *in vitro* stimulation of CD8 cells with nonspecific T cell stimulator PMA and ionomycin (PMI). The expression of exhaustion marker PD-1 and the negative checkpoint receptor TIGID also did not show statistical significance or correlation with the cytotoxic activity of CD8 cells from each individual animal (data not shown).

### DART Molecule-Mediated Activity Measured by Modified Quantitative Viral Outgrowth Assay (QVOA)

We sought to determine whether the 48-hour treatment with DART molecules would inhibit the number of infected cells responsible for cell-to-cell transmission of replication competent virus. To do so, we modified the Quantitative Viral Outgrowth Assay (QVOA) assay (9). Given the limited numbers of primary RM CD4+ T cells available from SHIV.CH505.375H-infected animals, we utilized the A66 cells as feeder cells. Forty-eight hours following incubation of reactivated SHIV-infected RM CD4+ T cells and autologous RM CD8+ T cells in the absence or presence of DART molecules, the cell cultures were washed to remove DART molecules. The high dissociation rate constants of the HIVxCD3 DART molecules and rhesus CD3-epsilon (5.2-5.8 x 10^-3^ s^-1^) and monovalent binding of anti-CD3 arm to CD3, which is does not have an avidity component, allows efficient elimination of DART molecules from cultures by three washing steps. Following the washed A66 feeders (1x10^6^ per well) were added. Cells were passaged every two to three days with fresh media without DART molecules (**Supplementary Figure 1A**). Culture supernatants from each condition were collected at day 9 and the amount of virus recovered was determined by measuring supernatant levels of SIV Gag p27 by ELISA. Increases in supernatant p27 levels occurred with 8 of 9 cultures that contained reactivated SHIV-infected RM CD4+ T cells alone (in the absence of autologous RM CD8 cells or DART molecules), which indicates presence of residual SHIV-infected RM CD4+ T cells capable to transmit replication-competent virus (**Supplementary Figure 1B**).

**Figure 5:**
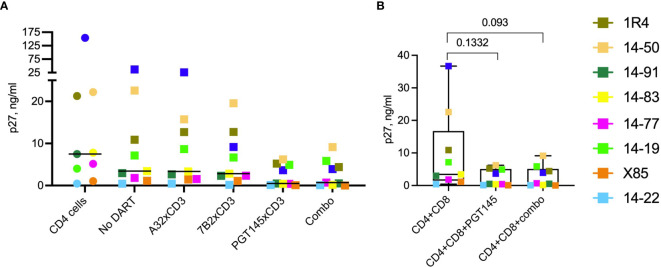
Effect of DART molecules on virus propagation. Cultures of reactivated SHIV-infected RM CD4+ T cells alone (CD4 cells) or mixed with autologous RM CD8+ T cells and incubated in the absence (No DART) or presence of DART molecules for 48 hours. DART molecules were then washed away and the cultures were mixed with A66 feeder cells to allow virus propagation. On day 9 cultures supernatants were analyzed for SIV Gag p27 levels by ELISA **(A)**. The horizontal lines represent the median values. **(B)** Statistical comparison between the indicated groups was performed using the Wilcoxon test at a significance level of 0.05. Combo is a combination of A32, 7B2 and PGT145 DART molecules.

As shown in **Figure 5A**, on Day 9 the median p27 level in the supernatants of cultures with reactivated SHIV-infected RM CD4 cells and autologous RM CD8 cells in the absence of DART molecules (*No DART*) declined to 3.4 ng/mL (range 0.4-35 ng/mL) compared to 7.5 ng/mL (range 0.4-154 ng/mL) detected in the cultures of reactivated SHIV-infected RM CD4+ T cells alone (*CD4 cells*). Compared to observed reduction in p27 in cultures with CD8 cells (*No DART*), single 48-hour treatment with A32 DART molecule reduced the levels of p27 in 2 of 9 cultures (median p27 3.4 ng/mL, range 0.4-28 ng/mL), with 7B2 DART molecule in 3 of 9 cultures (median p27 2.8 ng/mL, range 0.4-20 ng/mL). The minor reductions in p27 levels in cultures treated with A32 and 7B2 DART molecules was not significant compared to p27 levels observed in cultures with CD8 cells alone. Treatment with PGT145 DART molecule reduced supernatant p27 in 9 of 9 cultures with a median of 0.5 ng/mL (range 0.04-8 ng/mL). The triple DART combination reduced supernatant p27 levels in 9 of 9 cultures similar to PGT145 DART alone demonstrating a median p27 level of 0.7 ng/mL (range 0.04-12 ng/mL) (**Figure 5A**). The ranked ability to inhibit the virus propagation was PGT145 > 7B2 > A32. Cultures from animal 14-97 demonstrated the greatest reduction in virus recovery; supernatant p27 levels were 154 ng/mL for the reactivated SHIV-infected RM CD4 cells alone, which declined to 35 ng/mL following addition of autologous RM CD8 cells (*No DART*), which further declined to 3.6 ng/mL following addition of PGT145 DART molecule or triple DART molecule combination (**Figure 5A**). The average reduction in virus recovery mediated by the addition of PGT145 or combination of DART molecules to the cultures with CD4 and CD8 cells was 8-fold, but the difference was not statistically significant (**Figure 5B**).

### Assessment of Cytokine Release Concomitant With Cytolytic Activity

We measured *in vitro* cytokine production when cultures of primary reactivated SHIV-infected RM CD4+ T cells alone or mixed with autologous RM CD8+ T cells were incubated without (*No DART*) or with DART molecules for 48 hours. Cytokines measured included IL-1b, IL-6, IL-8, IL-12p40, IL-18, GM-CSF, IFN-γ and TNF-α (**Supplementary Figure 1**). None of the cytokines were detected when reactivated SHIV-infected RM CD4+ T cells were incubated alone. The addition of individual DART molecules or DART combination without or with autologous CD8+ T cells did not change production of IL-1b, IL-6, IL-8, IL-12p40, IL-18 cytokines (**Supplementary Figure 1A**). Low levels of GM-CSF, IFN-g and TNF-a (median <16pg/mL, <2pg/mL, and <4pg/mL, respectively) were detected following incubation of SHIV-infected RM CD4+ T cells with individual DART molecules or their combination. Incubation of SHIV-infected RM CD4+ T cells with autologous RM CD8+ T cells in absence of DART molecules resulted in production of GM-CSF, IFN-γ and TNF-α with median 4pg/mL, 15pg/mL, and 5pg/mL, respectively. The addition of DART molecules to CD4+CD8 autologous mixture had no or minimal effect on production of GM-CSF, IFN-γ and TNF-α (**Supplementary Figure 1A**). These small increases in cytokines did not correlate with the cytotoxic activity exhibited by these cultures (**Supplementary Figure 1B** **).**

## Discussion

Eradication of persistently infected cells is key to a functional cure of HIV infection. While ART treatment can successfully prevent viremia and disease progression, it does not lead to elimination of the virus, which persists as a quiescent provirus in rare latent infected cells. Life-long ART, which is required to prevent rebound of viremia and return of disease, is a financial and social burden. Novel immunotherapy-based strategies to treat HIV-1 need to address viral diversity and leverage the immune system’s ability to more efficiently target the latent viral reservoir. One potential approach is the “shock and kill” strategy where infected cells harboring latent virus are reactivated with latency reversing agents (LRAs) and eliminated by immunotherapies.

In the prior study, administration of a mixture of three HIVxCD3 DART molecules (having A32, 7B2 and PGT145 anti-HIV-1 Env specificities) and AZD5582 (latency reversing agent) to SHIV.CH505.375H-infected RMs that had been on ART for ~3 months did not result in reduction of the viral reservoir (41). The lack of DART molecule-mediated clearance activity in this animal model is attributed to the small size of the viral reservoir in the SHIV-infected animals maintained on ART and the absence of detectable latency reversal in response to AZD5582 treatment. The question persisted on whether DART molecules could engage the CD3-expressing effector cells of the animals to mediate target killing of reactivated Env-expressing SHIV-infected CD4+ T cells.

We, therefore, sought to confirm that DART molecules were capable of engaging primate effector cells and exerting an antiviral effect. We utilized primary cells isolated from the SHIV.CH505.375H-infected animal studied in Dashti et al. (41) and developed an *ex vivo* autologous system to assess DART molecule-mediated activities. We focused our work on the clearance of infected cells and utilized CD4+ T cells isolated from RMs at the peak of viremia (week 2 post infection), when the highest frequency of circulating infected cells expressed is expected and could be targeted for clearance. In order to assess whether DART molecules can recruit CD8 cells post ART treatment, we used autologous CD8+ T cells isolated from animals 20 weeks post ART initiation, when viremia was below the level of detection (47–49). According to our data, CD8+ T cells isolated from the RMs at this time may have only partially regained their cytotoxic function *in vivo*; under these conditions, the RM CD8+ T cells may not fully recapitulate the functionality of CD8+ T cells in PLWH who are maintained on ART before they can enroll in the clinical trials that evaluate the potency of these molecules.

Our data demonstrate that HIVxCD3 DART molecules were able to redirect RM CD8+ T cells to kill reactivated SHIV.CH505.375H-infected RM CD4+ cells and to reduce the level of replication competent virus released from the reactivated SHIV-infected RM CD4+ cells. The PGT145 DART molecule was more active than the 7B2 or A32 DART molecules, which is consistent with the ranked activities of these DART molecules in redirecting human CD8+ T cells to kill CH505 HIV-1 infectious molecular clone-infected human CD4+ cells. The PGT145 DART molecule, which is based on a broadly neutralizing antibody, is highly active toward CD4 cells infected by a more limited set of HIV-1 isolates than the 7B2 and A32 DART molecules, which are based on non-neutralizing, broadly reactive antibodies that recognize highly conserved epitopes in HIV-1 Env (34). We hypothesized that a combination of DART molecules based on a broadly neutralizing Ab (PGT145) and 2 non-neutralizing Abs (A32 and 7B2) can cover a broader spectrum of HIV-1 envelope conformations that are expressed on the membrane of the infected cells and upon reactivation of the latent virus (51–54). The principal finding of this study is that the triple DART molecule combination mediated optimal *ex vivo* redirected killing activity and inhibited propagation of replication competent virus. The combination of DART molecules demonstrated increase in killing in 4 of 9 animals, although the difference did not reach statistical significance compared to the PGT145 DART alone. Thus, the triple combination of DART molecules administered to the SHIV.CH505.375H-infected rhesus monkeys had potential to reduce the size of the virus reservoir if the co-administered LRA had generated targets for the DART molecules by reactivating latent infected cells to express HIV-1 Env on their surface.

Our data suggests that HIVxCD3 DART molecules have the potential to mediate elimination of the viral reservoir by cytotoxic CD8+ T cells after latency reversal. The effectiveness of viral eradication strategies requires both potent LRAs and cell-mediated cytotoxicity. Future studies designed to eradicate the viral reservoir in chronic infection should be powered to take in consideration the individual variability in the level of exhaustion and susceptibility to activation of the infection-induced immune responses.

## Data Availability Statement

The original contributions presented in the study are included in the article/**Supplementary Material**. Further inquiries can be directed to the corresponding author.

## Ethics Statement

The animal study was reviewed and approved by the Institutional Animal Care and Use Committee (IACUC) of Emory University and Yerkes National Primate Research Center.

## Author Contributions

MT, GF, and DM designed the experimental procedures. MT, AD, and KF conducted the experiments. SJ analyzed the data. JN generated the DART molecules. MT, GF, JN, and DM wrote the manuscript. AC and GS provided critical review of the results. All authors contributed to the article and approved the submitted version.

## Funding

This work was supported by Collaboratory of AIDS Researchers for Eradication (CARE), a Martin Delaney Collaboratory program; NIAID, National Institute of Neurological Disorders and Stroke, National Institute on Drug Abuse, and National Institute of Mental Health grant 1UM1AI126619-01, and NIH NIAID P01 grant AI120756. MT was supported by the NIH Ruth L. Kirschstein National Research Service Award (5T32AI007392). Work was also supported by federal funds from NIAID, NIH, Dept. of Health and Human Services under Contract No. HHSN272201500032C. Research was also supported by the Emory Consortium for Innovative AIDS Research in Nonhuman Primates (UM1 AI124436), the Yerkes National Primate Research Center (P51 OD011132), and the Translational Virology and Reservoir Cores of the Center for AIDS Research at Emory University (P30 AI050409). The content is solely the responsibility of the authors. The funders had no role in study design, data collection and analysis, decision to publish, or preparation of the manuscript.

## Conflict of Interest

JN is employed by MacroGenics and owns MacroGenics stock. JN, BH, and GF have pending patents on some of the molecules.

The remaining authors declare that the research was conducted in the absence of any commercial or financial relationships that could be construed as a potential conflict of interest.

## Publisher’s Note

All claims expressed in this article are solely those of the authors and do not necessarily represent those of their affiliated organizations, or those of the publisher, the editors and the reviewers. Any product that may be evaluated in this article, or claim that may be made by its manufacturer, is not guaranteed or endorsed by the publisher.

## Supplementary Material

Supplementary Figure 1 Modified quantitative viral outgrowth assay (QVOA). **(A)** Transmission of infection from reactivated SHIV-infected RM CD4+ T cells to A66 feeder cells. Primary CD4+ T cells from SHIV.CH505.375H-infected RMs were isolated and activated *in vitro* for 24 hours with anti-CD2/CD3/CD28 antibodies. The activated SHIV-infected RM CD4+ T cells were cultured alone or with autologous RM CD8+ cells for 48 hours in the absence or presence of DART molecules. On Day 4 the DART molecules were washed off and feeder A66 cells were added. Cells were split and media changed every 2-3 days. Supernatants collected at Day 4 and Day 9 were analyzed for SIV Gag p27 levels by ELISA to determine the amounts of SHIV virus that was produced. **(B)** p27 levels (ng/mL) in supernatants collected from cultures of activated SHIV-infected RM CD4+ T cells incubated in the absence of autologous CD8 cells or DART molecules. Each symbol indicates an individual animal. Animal 14-97 is graphed separately due to the difference in scare supernatant p27 level.

Supplementary Figure 2 Cytokine release concomitant with cytolytic activity. Cultures of primary reactivated SHIV-infected RM CD4+ T cells alone or mixed with autologous RM CD8+ T cells were incubated without (No DART) or with DART molecules for 48 hours. Cytokines measured in supernatants included IL-1b, IL-6, IL-8, IL-12p40, IL-18, GM-CSF, IFN-γ and TNF-α. Each symbol represents an individual animal; circles represent supernatants from CD4 cells and squares represent supernatants from mixtures of CD4 + CD8 cells. Limit of detection was set by the manufacturer at 1.6 pg/ml. (**C**) Statistical correlation between killing of infected cells by autologous CD8 cells in absence of DART molecules (refer to **Figure 4B**) and levels of GM-CSF, IFN-γ or TNF-α using two-tailed Pearson correlation coefficient with 95% confidence interval.
