# Supplementary material for: Elimination of SHIV Infected Cells by Combinations of Bispecific HIVxCD3 DART® Molecules
Source: Front Immunol. 2021 Aug 13;12:710273. doi: 10.3389/fimmu.2021.710273 (PMC8415083; doi:10.3389/fimmu.2021.710273)
Supplement: Supplementary Figure 1 — Modified quantitative viral outgrowth assay (QVOA). (A) Transmission of infection from reactivated SHIV-infected RM CD4+ T cells to A66 feeder cells. Primary CD4+ T cells from SHIV.CH505.375H-infected RMs were isolated and activated in vitro for 24 hours with anti-CD2/CD3/CD28 antibodies. The activated SHIV-infected RM CD4+ T cells were cultured alone or with autologous RM CD8+ cells for 48 hours in the absence or presence of DART molecules. On Day 4 the DART molecules were washed off and feeder A66 cells were added. Cells were split and media changed every 2-3 days. Supernatants collected at Day 4 and Day 9 were analyzed for SIV Gag p27 levels by ELISA to determine the amounts of SHIV virus that was produced. (B) p27 levels (ng/mL) in supernatants collected from cultures of activated SHIV-infected RM CD4+ T cells incubated in the absence of autologous CD8 cells or DART molecules. Each symbol indicates an individual animal. Animal 14-97 is graphed separately due to the difference in scare supernatant p27 level. [file DataSheet_1.pdf]

SUPPLEMENTARY MATERIALS

Elimination of SHIV infected cells by combinations of bispecific HIVxCD3 DART® molecules

Authors:

Marina Tuyishime[1,*], Amir Dashti[2], Katelyn Faircloth[1,#], Shalini Jha[1], Jeffrey L. Nordstrom[3], Barton F. Haynes[4,5,6], Guido Silvestri[2], Ann Chahroudi[2,7,8], David M. Margolis[9,10,11,12], Guido Ferrari[1,13].

Affiliations:

[1] Department of Surgery, Duke University Medical Center, Durham, North Carolina, USA.

[2] Department of Pediatrics, Emory University, Atlanta, GA 30322, USA.

[3] MacroGenics, Rockville, Maryland 20850, USA.

[4] Duke Human Vaccine Institute, Durham, NC, USA.

[5] Department of Medicine, Duke University Medical Center, Durham, NC, USA.

[6] Department of Immunology, Duke University Medical Center, Durham, NC, USA.

[7] Yerkes National Primate Research Center, Emory University, Atlanta, GA 30329, USA.

[8] Center for Childhood Infections and Vaccines of Children's Healthcare of Atlanta and Emory University, Atlanta, GA 30322, USA

[9] University of North Carolina (UNC) HIV Cure Center, University of North Carolina at Chapel Hill, Chapel Hill, NC, United States

[10] Department of Medicine, University of North Carolina at Chapel Hill, Chapel Hill, NC, United States

[11] Department of Microbiology and Immunology, University of North Carolina at Chapel Hill, Chapel Hill, NC, United States

[12] Department of Epidemiology, University of North Carolina at Chapel Hill, Chapel Hill, NC, United States

[13] Department of Molecular Genetics and Microbiology, Duke University Medical Center, Durham, NC, USA.

* Corresponding author, marina.tuyishime@duke.edu

# Currently research analyst at City of Hope, kfaircloth@coh.org.

Short title: DART-Mediated Killing of SHIV-Infected Cells

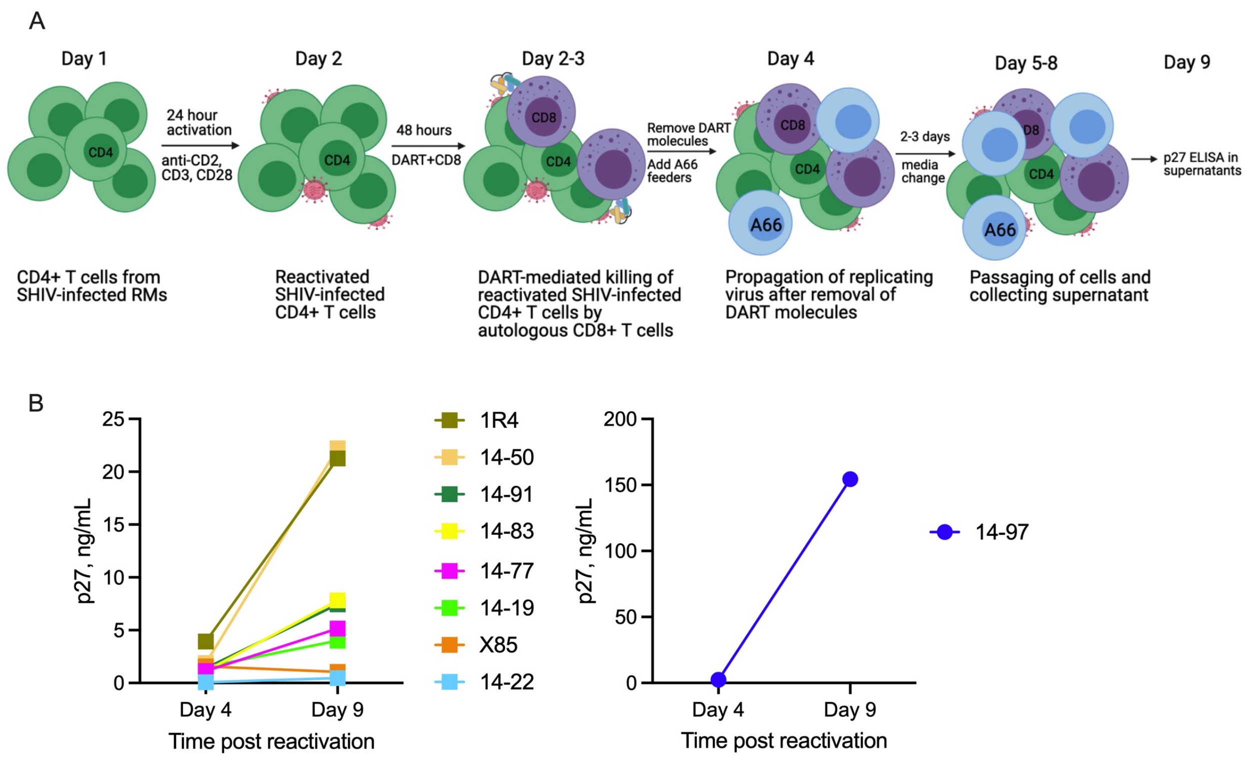

Figure S1. Modified quantitative viral outgrowth assay (QVOA). **(A)** Transmission of infection from reactivated SHIV-infected RM CD4+ T cells to A66 feeder cells. Primary CD4+ T cells from SHIV.CH505.375H-infected RMs were isolated and activated *in vitro* for 24 hours with anti-CD2/CD3/CD28 antibodies. The activated SHIV-infected RM CD4+ T cells were cultured alone or with autologous RM CD8+ cells for 48 hours in the absence or presence of DART molecules. On Day 4 the DART molecules were washed off and feeder A66 cells were added. Cells were split and media changed every 2-3 days. Supernatants collected at Day 4 and Day 9 were analyzed for SIV Gag p27 levels by ELISA to determine the amounts of SHIV virus that was produced. (**B**) p27 levels (ng/mL) in supernatants collected from cultures of activated SHIV-infected RM CD4+ T cells incubated in the absence of autologous CD8 cells or DART molecules. Each symbol indicates an individual animal. Animal 14-97 is graphed separately due to the difference in scare supernatant p27 level.

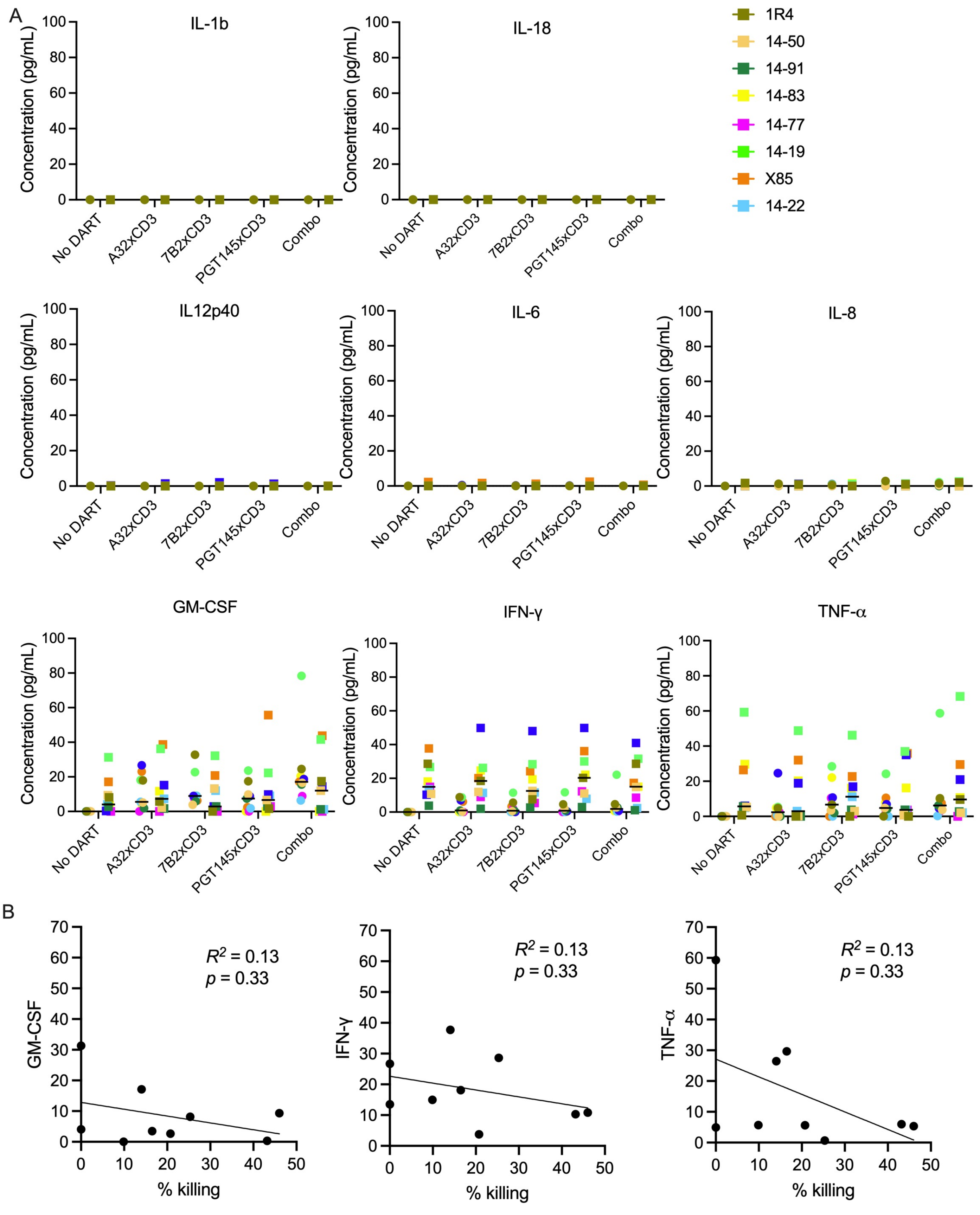

Figure S2. Cytokine release concomitant with cytolytic activity. Cultures of primary reactivated SHIV-infected RM CD4+ T cells alone or mixed with autologous RM CD8+ T cells were incubated without (No DART) or with DART molecules for 48 hours. Cytokines measured in supernatants

included IL-1b, IL-6, IL-8, IL-12p40, IL-18, GM-CSF, IFN-γ and TNF-α. Each symbol represents an individual animal; circles represent supernatants from CD4 cells and squares represent supernatants from mixtures of CD4 + CD8 cells. Limit of detection was set by the manufacturer at 1.6 pg/ml. (**C**) Statistical correlation between killing of infected cells by autologous CD8 cells in absence of DART molecules (refer to **Fig. 4B**) and levels of GM-CSF, IFN-γ or TNF-α using two-tailed Pearson correlation coefficient with 95% confidence interval.
